# Diversity Distribution, Driving Factors and Assembly Mechanisms of Free-Living and Particle-Associated Bacterial Communities at a Subtropical Marginal Sea

**DOI:** 10.3390/microorganisms9122445

**Published:** 2021-11-27

**Authors:** Huatao Yuan, Tangcheng Li, Hongfei Li, Cong Wang, Ling Li, Xin Lin, Senjie Lin

**Affiliations:** 1 State Key Laboratory of Marine Environmental Science, College of Ocean and Earth Sciences, Xiamen University, Xiamen 361102, China; 22320170154955@stu.xmu.edu.cn (H.Y.); tchli@stu.edu.cn (T.L.); lhf612@126.com (H.L.); cong@stu.xmu.edu.cn (C.W.); lingli@xmu.edu.cn (L.L.); xinlin@xmu.edu.cn (X.L.); 2 Department of Marine Sciences, University of Connecticut, Groton, CT 06340, USA

**Keywords:** marine bacterioplankton, spatial variability, particle associated, community assembly, iCAMP-based null model, neutral community model

## Abstract

Free-living (FL) and particle-associated (PA) bacterioplankton communities play critical roles in biogeochemical cycles in the ocean. However, their community composition, assembly process and functions in the continental shelf and slope regions are poorly understood. Based on 16S rRNA gene amplicon sequencing, we investigated bacterial communities’ driving factors, assembly processes and functional potentials at a subtropical marginal sea. The bacterioplankton community showed specific distribution patterns with respect to lifestyle (free living vs. particle associated), habitat (slope vs. shelf) and depth (surface vs. DCM and Bottom). Salinity and water temperature were the key factors modulating turnover in the FL community, whereas nitrite, silicate and phosphate were the key factors for the PA community. Model analyses revealed that stochastic processes outweighed deterministic processes and had stronger influences on PA than FL. Homogeneous selection (Hos) was more responsible for the assembly and turnover of FL, while drift and dispersal limitation contributed more to the assembly of PA. Importantly, the primary contributor to Hos in PA was Gammaproteobacteria:Others, whereas that in FL was Cyanobacteria:Bin6. Finally, the PICRUSt2 analysis indicated that the potential metabolisms of carbohydrates, cofactors, amino acids, terpenoids, polyketides, lipids and antibiotic resistance were markedly enriched in PA than FL.

## 1. Introduction

Microbial communities are highly diverse and play critical roles in biogeochemical cycling cycles across ecosystems, which are fundamental in maintaining climate and ecosystem stability [1,2,3]. In the marine environment, bacteria modulate carbon and nutrient cycling [4] and bacterial community composition is closely related to carbon export [5,6]. Bacterioplankton in the ocean can be classified into two groups based on their lifestyles: Free living (FL) and particle associated (PA) [7]. PA microbes rely on particulate organic matter (POM) as attachment substrate, which constitutes the hotspot microenvironment for the ecological process of marine microorganisms and biogeochemical cycle [8,9]. Indeed, particles of variable sizes, chemical compositions and physical properties conform to the microspatial architecture that structures the microbial environment, which differs from the bulk surrounding seawater in their chemistry and physics [4,10]. There are many different types of particles, such as living or dead protist cells, zooplankton and fish fecal pellets [4]. The patterns of bacteria diversity are known to vary greatly with the size of the particle to which the microbes are attached, and with the composition of the particles [11]. Genetically, PA microbes are very different from FL microbes. Typical PA microorganisms often have large or variable genomes and respond quickly to changing environments, are copiotrophic and are suited to living in nutrient-rich and variable marine environments [12]. By contrast, typical FL microorganisms tend to have small and streamlined genomes and are adapted to inhabiting relatively stable oligotrophic oceanic environments [12]. So far, many studies have shown differences in the diversity and taxonomic composition of free-living vs. particle-associated microbial communities in various environments, including lakes [13,14,15], rivers [16,17,18], estuaries and coastal oceans [11,19,20,21,22,23,24,25] and open oceans [26,27,28,29]. These studies showed that the similarity or difference between the free-living and particle-associated microbial communities strongly varies with the sampling site. However, most of these studies to date have considered only community structure, not community assembly.

One of the fundamental issues in microbial ecology is to identify and quantify ecological processes that drive bacterial community assembly in aquatic environments [22,30,31,32]. Community assembly has classically been classified as deterministic based on niche theory [33] and stochastic processes based on neutral theory [34], which jointly determine microbial biogeography and vary in their relative contribution to community assembly over different temporal and spatial scales [31]. To unify the niche and neutral perspectives on governing community structure, community assembly can be partitioned into four major processes: Selection, dispersal, speciation or diversification and ecological drift [35,36,37]. To quantify the relative importance of ecological processes in controlling microbial community diversity and succession, a framework to quantitatively infer community assembly mechanisms by phylogenetic bin-based null model analysis (iCAMP) was developed [38]. This improved the performance substantially with higher precision, sensitivity, specificity and accuracy than previous approaches. Importantly, iCAMP quantifies the relative importance of different ecological processes underlying community diversity and dynamics based on individual phylogenetic groups (bins) rather than the entire community, when compared with QPEN (Quantifying assembly Processes based on Entire-community Null model analysis) [39,40].

Moreover, iCAMP can provide information on the relative importance of different ecological processes in individual lineages (bins) [38]. Under this framework, selections are represented mostly by environmental correlations. Under variable environmental conditions, high variation in community structure could exist, which is referred to as variable selection (heterogeneous selection; Table 1). Under similar environmental conditions, little variation in community structure or species/compositional turnover is expected (homogeneous selection; Table 1). Dispersal refers to the movement of individuals between local communities, which we consider a stochastic process as microbial dispersal is predominantly passive [32]. Drift refers to stochastic changes with respect to species identity in the relative abundances of different species within a community over time due to the inherent random processes of birth, death and reproduction [32,35,37]. Speciation or diversification refers to the emergence of new species within a local community through evolutionary processes. In the marine environment, analysis of bacterioplankton community structure across longitudinal scales in the oligotrophic South Pacific Gyre showed homogeneous environmental selection dominates microbial community assembly [29]. By contrast, studies in the Chinese coastal bays and the East China Sea showed that stochastic processes outweighed deterministic processes in microbial community assembly [41,42]. In addition, previous research in bacterioplankton assembly also took into consideration the influence of seasonality [43,44]. These studies suggest that microbial community assembly processes are not conserved between different marine ecosystems and highlight the need for biome-specific investigations.

The continental shelf and slope represent a dynamic gradient of terrestrial influences as converging zones of terrestrial currents and ocean basins [45]. There occur intense land-sea material exchange, energy transfer and the interaction process between human activities and the natural environment, which play an important role in coupling biogeochemical processes between continents and oceans [46]. Biogeochemical cycles in these regions are closely related to microbial community composition [3]. However, the community assembly mechanisms which underpin microbial community structure in the continental shelf and the continental slope are not well understood.

This study was carried out across the continental shelf and slope in the northern South China Sea, a subtropical marginal sea surrounded by continents, to unravel spatial variability, functions, and processes of free-living and particle-associated bacterial communities. We aim at characterizing the bacterioplankton communities in various pico- to micro- size fractions (free living and particle associated) and describing their variability along horizontal (between the continental shelf and slope) and vertical (from surface waters down into the deep chlorophyll maxima and a deeper layer) gradients in the northern South China Sea. We used high-throughput sequencing to evaluate the diversity and community composition of the bacterioplankton communities and determine the spatial patterns and main drivers of bacterioplankton diversity, community composition, assembly process and potential metabolic functions.

## 2. Materials and Methods

### 2.1. Sample Collection and Environmental Variables

**Figure 1:**
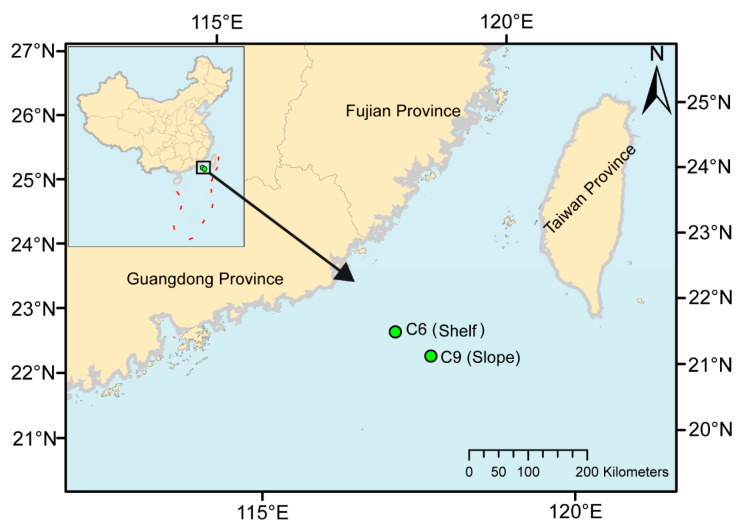
Location of the study area in northern South China Sea map showing the stations sampled. Sampling was carried out at Station C6 (117.46° E, 22.13° N) at the surface and DCM, which is on the continental shelf with a depth of 77 m; and Station C9 (117.99° E, 21.69° N) at the surface, DCM and Bottom, which is on the continental slope with a depth of 1369 m.

To examine the spatial dynamics of bacterial communities of the costal shelf-slope region, sampling was carried out at the shelf station C6 (117.46° E, 22.13° N) and the slope station C9 (117.99° E, 21.69° N) in the South China Sea (Figure 1). South China Sea is a marginal sea in the Western Pacific Ocean as it is surrounded by China mainland, Indo-China Peninsula, greater Sunda Islands and the Philippines Islands. Sampling depths included the surface, deep chlorophyll maximum (DCM), which was the depth of 45 m at C6 and 75 m at C9, and for C9 also a deep depth at 150 m (Bottom). The sample codes, locations and study area description are presented in Table S2. For each sample, 40–50 L seawater from more than three CTD rosettes (each with a capacity of 12 L) was first filtered through a 200 μm bolting cloth to remove large plankton. Two size fractions were obtained by sequential filtration of 200 μm prefiltered water through 3.0 μm and 0.22 μm using 142-mm diameter polycarbonate membranes (Millipore, Billerica, MA, USA) completed within 20 min. Bacteria in the 0.22~3.0 μm size fraction are considered free-living, and those in size fraction 3.0~200 μm are considered particle associated [47]. Finally, the sample-bearing filters were each placed into a 2-mL tube containing 1 mL lysis buffer (0.1 M EDTA and 1% SDS) and stored at liquid nitrogen until DNA extraction. Temperature, salinity and depth were measured using a CTD profiler. A series of nutrients were measured using continuous-flow Technicon AA3 AutoAnalyzer. The detection limits of nitrate + nitrite (NO_3_^−^ + NO_2_^−^), nitrite (NO_2_^−^), phosphate (PO_3_^−^) and silicate (Si^3^O_2_^−^) were 0.03 μmol L^−1^, 0.04 μmol L^−1^, 0.03 μmol L^−1^ and 0.05 μmol L^−1^, respectively.

### 2.2. DNA Extraction and Sequencing

Total DNA extraction was carried out using a CTAB protocol combined with Zymo DNA Clean & Concentrator kit (Zymo Research Corp, Irvine, CA, USA) as previously reported [48]. DNA concentrations were determined using a NanoDrop ND-2000 Spectrophotometer (Thermo Fisher Scientific, Carlsbad, CA, USA). The bacterial 16S rRNA genes were amplified using the primer pair 515F (5-GTGCCAGCMGCCGCGGTAA-3) and 806R (5-GGACTACHVGGGTWTCTAAT-3) [49]. The polymerase chain reaction protocol consisted of an initial denaturation at 98 °C for 30 s; followed by 30 cycles of denaturation at 98 °C for 10 s, annealing at 50 °C for 30 s and elongation at 72 °C for 60 s; and a final elongation step at 72 °C for 5 min. The PCR products were purified using Agencourt AMPure XP beads and eluted in the Elution buffer. The purified amplicons were quality-checked using the Agilent 2100 Bioanalyzer. The validated amplicon samples were sequenced on MiSeq (Illumina, San Diego, CA, USA) in a barcoded multiplex fashion, with more than 20 Mbp data output of each sample. The generated raw sequence data were deposited at the Sequence Read Archive of the NCBI, BioProject number PRJNA782430.

### 2.3. DNA Sequence Analysis and Function Inference

We used the QIIME2 pipeline (version 2020.2) to process the 16S rRNA gene [50]. The demultiplex sequences were uniformly trimmed to 250 bp (forward and reverse), and then were denoised to infer Amplicon Sequence Variants (ASVs) that were 462 bp in length using the DADA2 plugin with default settings [51]. Taxonomy was assigned to representative sequences using the Silva 138 Naive Bayes 515F/806R taxonomy classifier. All ASVs affiliated to archaea, chloroplasts and mitochondria, as well as singletons, were removed from the dataset. A phylogenetic tree was generated from representative sequences by aligning sequence fragments via MAFFT, masking ambiguous alignments and inferring a tree using the FastTree algorithm [52]. A rooted tree was created by putting the root at the midpoint of the farthest tips in the tree. To make samples comparable, the feature table was rarefied to a depth of 9000 sequences per sample. We estimated beta diversity using both the weighted and unweighted UniFrac distance in QIIME 2. In addition, PICRUSt2 was used to predict metagenomic functions based on the normalized feature tables [53].

### 2.4. Quantification of Community Assembly Processes 

To analyze the relative importance of various ecological processes, variation partitioning, null model and neutral community model (NCM) were performed. The variation partitioning approach was used to evaluate the relative influence of environmental factors, spatial variabilities and their coinfluences on community composition variations. To further elucidate community assembly processes, null model analysis was conducted. The governing processes were determined from the models of variable selection, homogeneous selection, dispersal limitation, homogenizing dispersal and undominated using weighted beta Net Relatedness Index (βNRI) in combination with Raup–Crick metric (RC) [38]. As shown in Table 1, for βNRI, values < −1.96 indicate homogeneous selection, whereas values > 1.96 indicate heterogeneous selection. The RC metric was used to partition the remaining parts with |βNTI| ≤ 1.96. Here, RC < −0.95 represents homogenous dispersal, RC > 0.95 represents dispersal limitation and |RC| ≤ 0.95 represents ecological drift (undominated). Null models were constructed using 999 randomizations, as described by Ning et al. [38].

Moreover, NCM [54] was also used to assess the potential contribution of stochastic processes to the assembly of bacterial communities by modeling and predicting the relationship between ASVs relative abundance and their occurrence frequency across the wider meta-community. The influences of drift (birth-death immigration process) and stochastic dispersal processes can be incorporated in this neutral model. The R^2^ values represent the goodness of fit to the NCM, and larger values of R^2^ indicate stochastic dispersal and ecological drifts are more important than selection in community assembly by the neutral model [54,55]. Model calculations were performed using the minpack.lm and HMisc packages in R [55,56].

### 2.5. Statistical Analyses 

**Figure 2:**
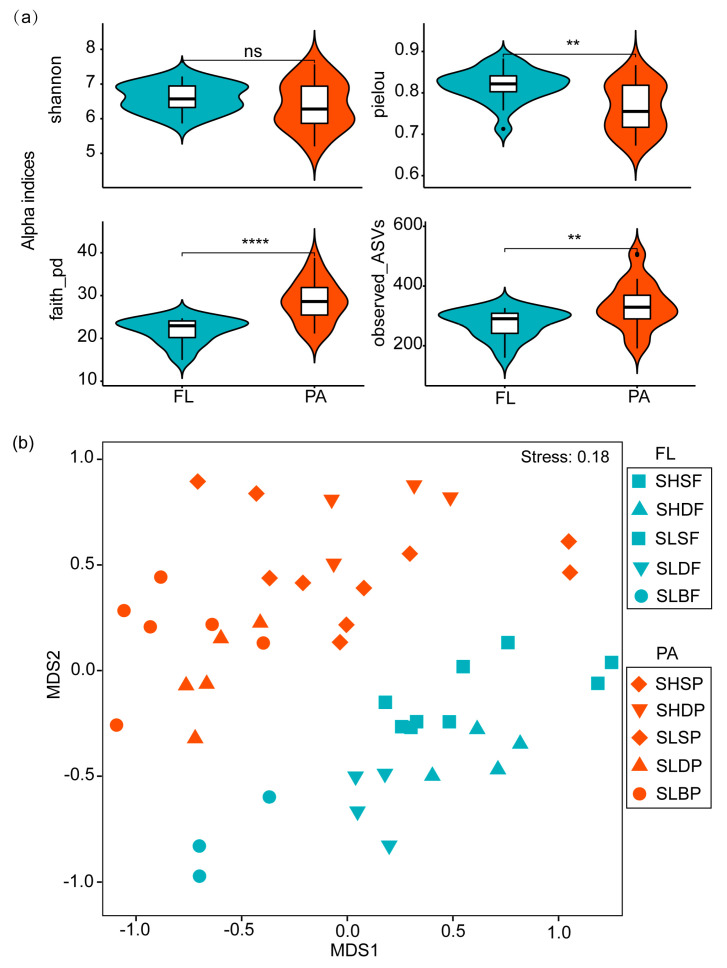
(**a**) Differences in alpha diversity between FL and PA communities. One-side significance based on bootstrapping test was expressed as **** *p* < 0.001; ** *p* < 0.05; ns > 0.05. (**b**) Two-dimensional NMDS ordination using Bray–Curtis indices of bacterioplankton communities. The samples were collected in June 2016 in MACRO cruise, and samples grouped by two size fractions (free living and particle associated), different stations (Slope and Shelf) and depth (Surface, DCM and Bottom). Site names: SL = Slope, SH = Shelf, S = Surface, D = DCM, B = Bottom, F = fre -living and P = particle associated (Table S2). The numbers of replicated samples in this figure are as follows: In FL, SHSF (*n* = 4), SHDF (*n* = 4), SLSF (*n* = 5), SLDF (*n* = 4), SLBF (*n* = 3); in PA, SHSP (*n* = 4), SHDP (*n* = 4), SLSP (*n* = 6), SLDP (*n* = 5), SLBP (*n* = 6).

All subsequent analyses were mainly carried out in R (v4.0.2). To investigate the significance of changes in microbial community composition between a priori groups of samples (grouped as shown in Figure 2b and Table S2), non-metric multidimensional scaling (NMDS) ordination analysis of similarity (ANOSIM) tests based on the Bray–Curtis distance were performed using the “vegan” package in R v4.0.2. Single-factor ANOVA and Tukey’s HSD test were performed to determine the significance of differences between each sample group with respect to environmental parameters, alpha indices and the relative abundances of specific bacterial taxonomic groups. The alpha diversity indices of bacterial communities, including Pielou, faith_pd, Shannon and Observed_ASVs, were calculated using PAST v3.0 [57]. Two-way analysis of variance (ANOVA) was used to test the significance of season, location and their interaction on environmental factors and alpha diversity indices using PAST v3.0 [57]. The relative abundance of dominant bacteria at different taxonomic levels was envisioned by “ggplot2′′ package in R v4.0.2. Mantel test was performed to evaluate the linkages between community structure of the planktonic groups and environmental parameters, which were visualized using “vegan” package. Bacterioplankton with significantly different abundances in different size-fractions were recognized by STAMP based on Welch’s *t*-tests with an adjusted false discovery rate (FDR) [58]. 

## 3. Results

### 3.1. Spatial Variations in Alpha and Beta Diversity

A total of 45 water bacterioplankton communities at different depths from the continental shelf and slope were analyzed based on the bacterial 16S rRNA genes (Table S2). Overall, 770,050 tags were obtained in all the samples, 9026 to 19,617 per sample (on average 17,112 tags per sample). After quality control and rarify, a total of 405,000 high-quality tags (average of 9000 tags per sample) were retrieved for taxonomic annotation of the bacterioplankton. 

As shown in Figure 2a and Figure S2, clear differences were observed between the particle-associated bacterial community (PA, >3 μm) and the free-living community (FL, 0.22~3.0 μm) in alpha diversity. The Shannon-Wiener and Pielou indices of PA were lower than that of FL, while Faith_pd and species richness (observed_ASVs) of PA was significantly greater relative to FL. For FL, the Faith-pd and species richness increased with increasing depth (S < D < B), whereas the Shannon and Pielou indices were the lowest at the surface and the highest at DCM. For PA, the Shannon, Pielou and Faith-id indices decreased with increasing depths, a reverse trend relative to FL (Figure S2), whereas the species richness had a similar trend to FL (Figure S2). The two-way ANOVAs with factors for habitat and depth interaction for the alpha diversity indices of bacterioplankton communities suggested depth was a major influencing factor for the Shannon, Pielou and Observed_ASVs, whereas location (station) was mainly responsible for the Faith_pd (Table S3).

NMDS ordination revealed that the bacterioplankton communities were grouped by size fraction (free living vs. particle associated), habitat (shelf vs. slope) and water depth (surface vs. DCM vs. Bottom) (ANOSIM R-value: 0.52 vs. 0.39 vs. 0.30, respectively) (Figure S3). Meanwhile, the variability of the bacterioplankton communities related to particle size (i.e., size-fractions) was represented by the factor “size-fraction”, the coastal to oceanic variability of the communities by the factor “station” and their vertical variability by the factor “depth”. The PERMANOVA test revealed statistically significant differences in bacterioplankton community structure due to all three factors (*p* < 0.001) (Table S1), yet the variability due to “size-fraction” (19%) and “station” (14%) was much higher than that explained by “depth” (6.9%). In addition, the community composition of the smaller size fraction was more variable between depths and stations (i.e., higher beta diversity) than those of the largest size fraction (Table S4).

More importantly, the samples of bacterioplankton formed ten groups that were significantly different for all the microbial communities in an NMDS ordination space built from ASVs-based Bray–Curtis distances (*p* = 0.001), and the global R values for the entire bacterioplankton among these groups were 0.7176 (Figure 2b and Table S5). However, SHSF vs. SHDF and SHSP, SLBF vs. SHSP and SHDP, SLDP vs. SLBP had no significant differences (*p* > 0.05) (Table S5). Overall, the mean Bray–Curtis distance between samples belonging to FL was lower than the mean distance between samples belonging to PA (Figure S2 and Table S5).

### 3.2. Spatial Variation of Bacterioplankton Community

**Figure 3:**
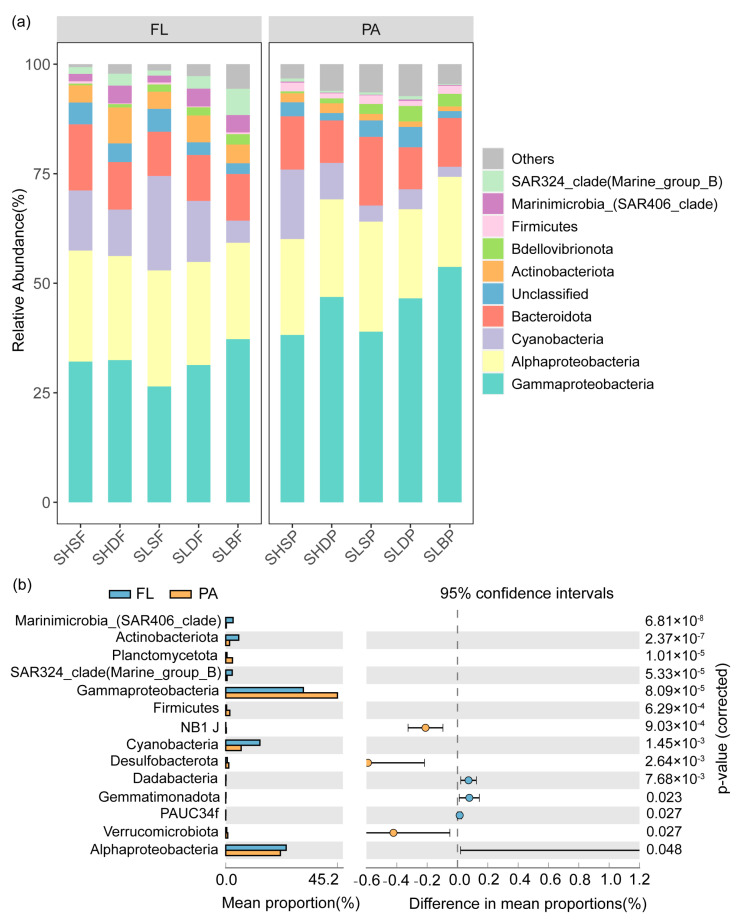
(**a**) Stacked bar chart showing relative abundance of the top 10 taxa during free-living and particle-associated communities. (**b**) Bacterioplankton phylums/classes with significantly different abundances between free-living and particle-associated communities.

Gammaproteobacteria (39.02%) was the dominant subphylum in all the microbial communities, followed by Alphaproteobacteria (23.14%), Bacteroidota (11.64%), Cyanobacteria (9.60%), Actinobacteria (3.20%), Bdellovibrionota (1.83%), SAR324_clade (Marine_group_B) (1.45%), Marinimicrobia_(SAR406_clade) (1.43%) and Firmicutes (1.06%) (Figure 3a). We further analyzed the compositions of the FL and PA communities. Significantly higher abundances of Marinimicrobia_(SAR406_clade), Actinobacteria, SAR324_clade (Marine_group_B), Alphaproteobacteria and Cyanobacteria were found in the FL community than in the PA community (Welch’s *t*-test, *p* < 0.05, Figure 3b). By contrast, the abundances of Gammaproteobacteria, Planctomycetota, Firmicutes, Verrucomicrobiota and Desulfobacterota were significantly higher in the PA than those in the FL community (Welch’s *t*-test, *p* < 0.05, Figure 3b).

Besides, we also found similar phylum/subphylum distribution patterns across water depth between the FL and the PA communities (Figure S4). For example, Cyanobacteria decreased quickly with increasing water depth. By contrast, the relative abundance of Marinimicrobia_(SAR406_clade), SAR324_clade (Marine_group_B), Bdellovibrionota and Gammaproteobacteria showed continuous increases with increasing depth. The relative abundance of Firmicutes, Bacteroidota, Gammaproteobacteria, Bdellovibrionota and Alphaproteobacteria were stable across all bacterioplankton communities, with no significant difference from each other (Figure S4). At the family level, the significantly higher abundances of Clade_I, SAR86_clade, Actinomarinaceae, Marinimicrobia_SAR406_clade and SAR324_calde (Marine_group_B) were found in the FL community than in the PA community (Welch’s *t*-test, *p* < 0.05, Figure S5). The relative abundances of Actinomarinaceae, Marinimicrobia_SAR406_clade and SAR324_clade(Marine_group_B) in the FL community showed continuous increases with increasing depth. However, The relative abundance of Clade_I showed continuous decreases with increasing depth in the FL and PA communities (Welch’s *t*-test, *p* < 0.05, Figure S5).

### 3.3. Environmental and Spatial Factors Influencing Bacterioplankton Community 

The comparisons of environmental factors related to size-fractions and location are shown in Figure S1. Seawater environment factors included nitrite, phosphate, silicate, nitrate, temperature and salinity. The depths we sampled at each station presented pronounced vertical physicochemical variation. Water temperature decreased with increasing depth, from 30.62 °C at the surface to 16.95 °C at the 150 m layer. Salinity and the concentrations of phosphate and nitrate showed a gradual increase with the lowest at surface water and the highest at the 150 m layer, while the concentration of nitrite fluctuated, with an overall decreasing trend, along the whole water column (Figure S1). 

**Figure 4:**
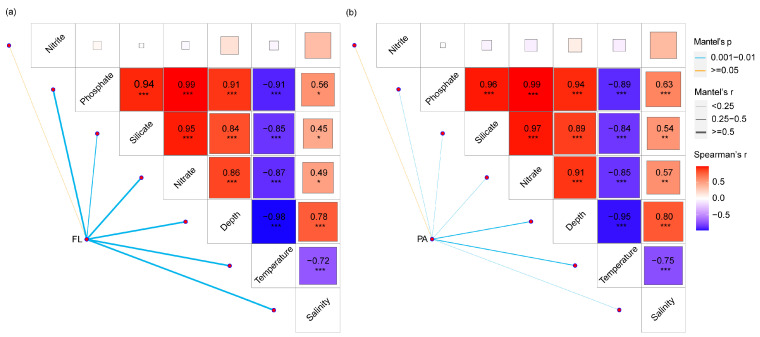
Environmental drivers of bacterioplankton community in continental shelf and slope regions evaluated by Mantel tests. Edge width corresponds to R value and edge color indicates statistical significance. Color gradient represents Spearman correlation coefficients between environmental variables. Asterisks indicate significance (* *p* < 0.05; ** 0.05 < *p* < 0.01; *** *p* < 0.01). (**a**) Free-living community. (**b**) Particle-associated community.

To explore factors influencing the community structure, the mantel test and VPA (Variance Partitioning Analysis) between community composition and environmental-spatial factors were analyzed. Results showed that salinity, temperature, nitrate, phosphate depth and silicate were significantly correlated with the bacterioplankton composition for all samples (Figure 4). For the FL community (Figure 4a), bacterioplankton exhibited significant correlations with salinity (r = 0.521, *p* = 0.01), nitrate (r = 0.540, *p* = 0.01), temperature (r = 0.675, *p* = 0.01), phosphate (r = 0.561, *p* = 0.01), depth (r = 0.705, *p* = 0.01) and silicate (r = 0.493, *p* = 0.01), but not for nitrite (r = −0.068, *p* = 0.725). Whereas for the PA community (Figure 4b), salinity (r = 0.204, *p* = 0.02), nitrate (r = 0.249, *p* = 0.03), temperature (r = 0.276, *p* = 0.01), phosphate (r = 0.239, *p* = 0.03), depth (r = 0.298, *p* = 0.01), silicate (r = 0.228, *p* = 0.03) showed significant correlations with the bacterioplankton communities, except for nitrite (r = 0.015, *p* = 0.399). These results indicated that bacterioplankton responded sensitively to environmental variations in the continental shelf and slope regions.

**Figure 5:**
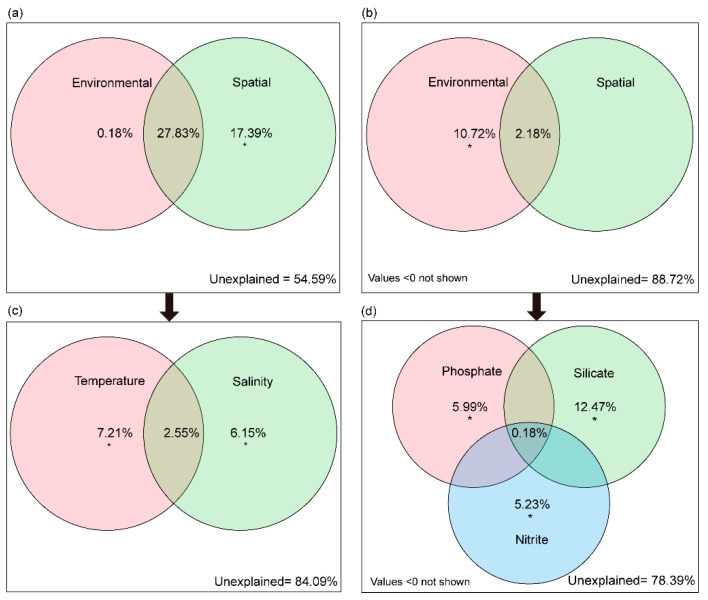
Venn diagrams of variation partitioning analysis showing the effects of spatial and environmental factors on the bacterioplankton communities. The percentage of variation explained by each factor, including unique, shared and unexplained is shown in corresponding positions in the diagram. When no numbers are shown, this matrix had no explanatory wer (zero or negative). Asterisks represent statistical significance: * *p* < 0.05, as estimated using canonical correspondence analysis. FL community (**a**,**c**); PA community (**b**,**d**).

Based on VPA, the conditional effects of environmental and spatial factors on the FL community were 0.18% and 17.39%, respectively (Figure 5a). Meanwhile, temperature and salinity were identified as the most important environmental factors shaping the FL community, with conditional effects of 7.21% and 6.15%, respectively (*p* < 0.05, Figure 5c). The conditional effect of environmental factors on the PA community was 10.72%, whereas spatial factors had no effect (Figure 5b). Moreover, phosphate, silicate and nitrite had significant effects on the PA community, and the conditional effects were 5.99%, 12.47% and 5.23%, respectively (*p* < 0.05, Figure 5d).

### 3.4. Community Assembly Mechanisms of Bacterioplankton

**Figure 6:**
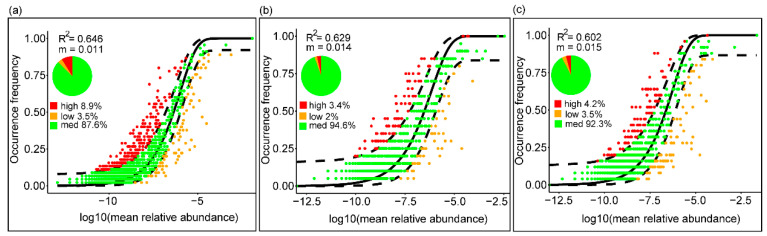
Fit of the neutral model for microbial community. The predicted occurrence frequencies for (**a**–**c**) bacterioplankton communities representing all, free-living and particle-associated samples, respectively. ASVs that occur more frequently than predicted by the model are shown in red while those that occur less frequently than predicted are shown in orange. Dashed lines represent 95% confidence intervals around the model prediction (black line).

To explore mechanisms underpinning the observed spatial patterns, the relative roles of niche and neutral processes in community assembly were analyzed. The Sloan neutral model was fitted to the data set to investigate the contribution of stochastic processes to free-living and particle-associated bacterioplankton community assembly. The neutral model explained a large fraction of the relationship between the occurrence frequency of ASVs and their relative abundance variations (Figure 6), with 64.6%, 62.9% and 60.2% of explained variance for all samples, the free-living and particle-associated communities. Furthermore, the R^2^ value ranged from 0.517 to 0.655, with the lowest in the group SHDP and the highest in SLSF (Figure 6, Table S6). In the same water layer, the neutral interpretation provided a better fit for the continental slope communities than for the continental shelf communities (Figure 6, Table S6), indicating that the influence of stochastic processes increased in the continental slope compared with the continental shelf. The majority of the ASVs were well predicted by the model, with values from 87.6% to 94.6% (Figure 6). Concurrently, small variations of the immigration rate *m* were observed in the whole community, the FL community and the PA community. The estimated migration rate (m value), a measure of the influence of dispersal on community composition, was 0.011, 0.014 and 0.015 for the whole community, the FL community and the PA community, respectively (Figure 6). The estimated migration rates tended to be higher in the PA than the FL, suggesting that the dispersal limitation had a stronger effect on the PA community than the FL community. 

#### 3.4.1. Effects of Lifestyle on Bacterioplankton Community Assembly

**Figure 7:**
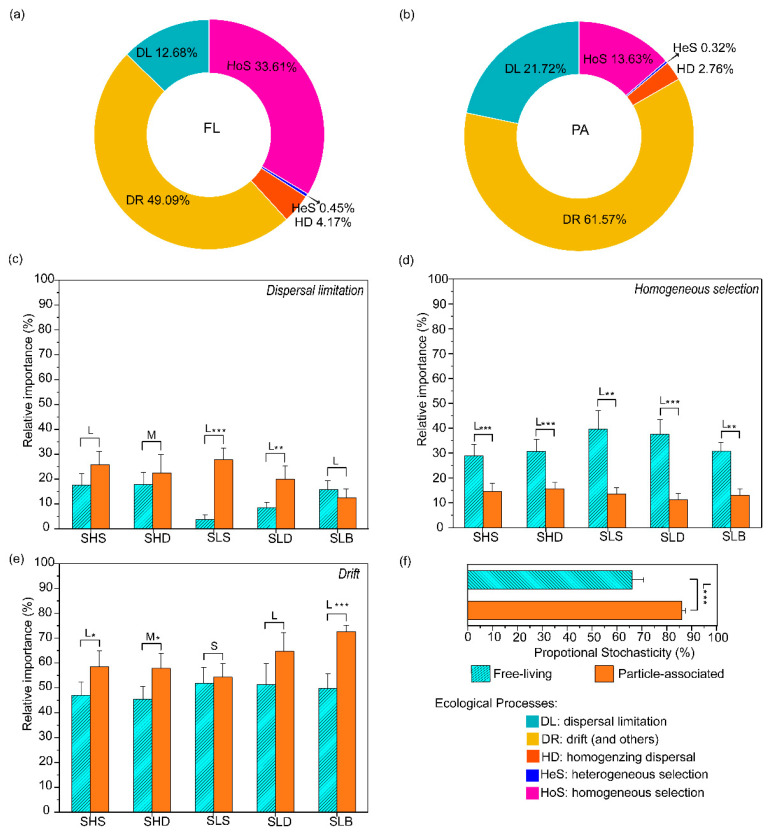
Doughnut and bar plots displaying the relative influences of assembly processes governing community turnover. (**a**) free-living community. (**b**) particle-associated community. (**c**) Changes of homogeneous selection under particle associated (orange bar) and free living (aqua bar). (**d**) Changes of dispersal limitation. (**e**) Changes of drift. (**f**) Stochasticity estimated by iCAMP under particle associated (orange bar) and free living (aqua bar). One-side significance based on bootstrapping test was expressed as *** *p* < 0.01; ** *p* < 0.05; * *p* < 0.1. *p* = 0.0061, 0.0000, 0.0139, 0.0000, 0.0000 in 5 different groups for homogeneous selection; *p* = 0.1129, 0.2884, 0.0000, 0.0330, 0.2595 for dispersal limitaton; *p* = 0.0962, 0.0706, 0.3861, 0.1067, 0.0000 for drift, respectively. L, M, S and N represented large (|d| > 0.8), medium (0.5 < |d| ≤ 0.8), small (0.2 < |d| ≤ 0.5) and negligible (|d| ≤ 0.2) effect sizes of size-fraction, based on Cohen’s d (the mean difference between particle associated and free living divided by pooled standard deviation). Data are presented as mean values ± SD. Error bars represented standard deviations. (**c**,**d**,**f**) *n* ≥ 3 biologically independent samples at each group.

The null model based on iCAMP analysis was applied to quantify the contribution of various ecological processes to FL and PA bacterioplankton community assembly. Furthermore, the relative contribution of the observed taxa divided into different groups (‘bins’) to various ecological processes was also analyzed. The null model results revealed that drift (and others), homogeneous selection and dispersal limitation were more dominant mechanisms shaping the bacterioplankton communities, with an average relative importance of 49.09~61.57%, 13.63~33.61% and 12.68~21.72% (Figure 7a,b), respectively. In contrast, heterogeneous selection and homogenizing dispersal only represented a small percentage of variation in the bacterioplankton communities, with an average relative importance of 0.32%~0.45% and 2.76%~4.17% (Figure 7a,b), respectively. The relative importance of dominant processes between FL and PA bacterioplankton was significantly different (Figure 7c–e). Since other processes had relatively low estimated relative importance (<4.17%), we primarily focused on the effects of homogeneous selection, dispersal limitation and drift on FL and PA bacterioplankton in subsequent analyses.

Overall, the PA community had higher relative importance of drift and dispersal limitation and lower relative importance of homogeneous selection, when compared with the FL community. Significant variations in different groups were observed (Figure 7c–e). The PA community showed significantly higher ratio of drift (Effect size: Cohen’s d = −2.19~−0.41, *p* < 0.05) and dispersal limitation (effect size: Cohen’s d = −6.56~0.94, *p* < 0.05), but lower ratio of homogeneous selection (effect size: Cohen’s d = 3.67~5.79, *p* < 0.05) than the FL community (Figure 7c–e). Drift (and others) contributed less to the community assembly with increasing water depth, however, the opposite trend was found in the PA community at the continental slope station (Figure 7e and Table S7). These results indicate that drift became more dominant in bacterioplankton community assembly with increasing water depth.

Dispersal limitation, which causes divergence in community composition because of a limited exchange of microbes [39], decreased quickly with increasing water depth in the PA community. In contrast, the relative contribute in the FL community continuously increased with increasing depth (Figure 7c and Table S7). Homogeneous selection had a relatively higher importance in the FL community as compared to the PA community. Regardless of whether the free-living or particle-associated community, homogeneous selection increased with increasing water depth in the continental shelf, but an opposite trend was noticed in the continental slope.

#### 3.4.2. Stochastic vs. Deterministic Bacterial Assembly

Based on the principle of the null models employed by iCAMP, the fractions of dispersal limitation, homogenizing dispersal and drift are largely considered stochastic [31]. Thus, the sum of their estimated relative importance can be used to estimate the stochasticity of community assembly. Based on iCAMP results, there showed a significantly higher ratio of stochastic processes in the particle-associated community in comparison with the free-living community (86.04% vs. 66.02%, Cohen’s d = 5.772, *p* < 0.001, Figure 7f), suggesting the bacterioplankton community assembly was even more stochastic for the PA community than the FL community. 

#### 3.4.3. Assembly Mechanisms across Different Phylogenetic Groups

In contrast to other approaches, iCAMP allows us to infer information on the relative importance of different ecological processes in individual lineages (bins). Based on the iCAMP analysis, the observed 2515 ASVs were divided into 94 phylogenetic bins. Relative abundances of different phyla in the FL community and PA community, Gammaproteobacteria:Others (20.48% vs. 19.09%) was the dominant phylum/class in the all microbial communities, followed by Cyanobacteria:Bin6 (13.80% vs. 6.16%), Gammaproteobacteria:Bin91 (11.41% vs. 26.30%), Bacterioidota (11.40% vs. 11.85%), Alphaproteobacteria:Others (10.79% vs. 14.55%) (Figure S6). We further analyzed their relative contributions to homogeneous selection (HoS), dispersal limitation (DL) and drift (DR) for the FL and PA communities (Figure S6). Higher relative contributions to HoS in the PA community than the FL community, including Alphaproteobacteria:Bin63, Gammaproteobacteria:Bin91, Gammaproteobacteria:Others, Alphaproteobacteria:Others, Planctomycetota, Bacteroidota and Actinobacteriota: Others. By contrast, SAR324_clade (Marine_group_B), Cyanobacteria:Bin6, Alphaproteobacteria:Bin69, Actinobacteriota:Bin30 had more contributions to HoS in the FL community. Besides, we also found that these phyla in the FL community had opposite contributions to Drift and dispersal limitation compared to homogeneous selection compared with the PA community. For example, Gammaproteobacteria:Others had more contributions to drift and dispersal limitation in the FL community than the PA community, whereas Cyanobacteria:Bin6 had more contributions to homogeneous selection in the FL subcommunity than the PA community (Figure S6).

### 3.5. Predicting Metabolic Functions That Might Underlie Changes between the FL and PA Community Structure

**Figure 8:**
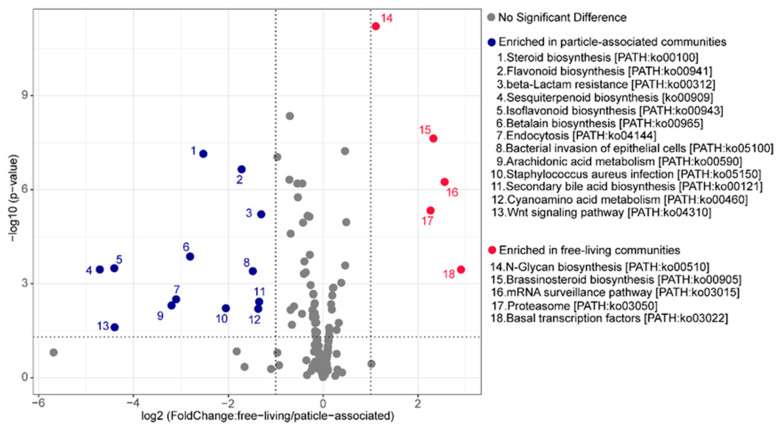
KEGG pathways predicted by PICRUSt2 to be enriched in the PA (blue) and FL (red) communities. Enriched pathways have an adjusted *p*-value < 0.05 and log2 fold change > 1 (twofold change), the metabolic pathways with no significant difference in PA and FL are indicated by gray circles.

Metagenome inference from the normalized feature tables was performed using PICRUSt2. The weighted nearest sequenced taxon index (NSTI) values ranged between 0.08–0.19 (average 0.12) for the PA communities and 0.1~0.16 (average 0.12) for the FL lineages, indicating that these ASVs were representative of the reference genome database used and could generate reasonable functional predictions. In this study, we inferred the metagenome of the communities using PICRUSt2, and searched for specific genes and metabolic pathways predicted to be differentially abundant between the PA and FL communities (Figure 8). As shown in Figure 8, more pathways were enriched in the PA community compared with the FL community, consistent with the generally larger genomes and richer metabolic capacities of particle-associated bacteria [59]. The enriched pathways in the PA community were related to secondary metabolites, such as steroid biosynthesis, secondary bile acid biosynthesis, sesquiterpenoid biosynthesis, flavonoid biosynthesis, isoflavonoid biosynthesis, arachidonic acid metabolism and betalain biosynthesis. The additional enriched pathways in the PA community were for *Staphylococcus aureus* infection, bacterial invasion of epithelial cells and endocytosis. Regarding nutrient metabolism, pathways we found included cyanoamino acid metabolism, which plays a vital role in the nitrogen metabolism of marine microbe [60]. Pathways related to antibiotic resistance were also significantly enriched in the PA community, such as beta-Lactam resistance. Only five pathways were enriched in the FL community compared with the PA community, consistent with their typically smaller genomes. These pathways were related to N-glycan biosynthesis, brassinosteroid biosynthesis, mRNA surveillance pathway, basal transcription factors and the proteasome.

## 4. Discussion

### 4.1. General Structure of Bacterioplankton Community Structure across Size Fraction, Water Depth and Station

As shown in Figure 2a,b and Table S1, clear differences were observed between the particle-associated bacterioplankton community (PA, >3 μm) and the free-living community (FL, 0.22~3 μm). Indeed, the Bray Curtis dissimilarity was highest between the FL and PA communities, followed by the difference with station, with the lowest variability observed between depth (Figure 2b and Figure S3, Table S1). Thus, lifestyle seems to be the most important driver of differential microbial community composition in the continental shelf and slope. As shown in Figure S2, the FL community was more diverse (Shannon indices) and even (Pielou indices) than the PA, although Shannon indices were not significantly different. These results are consistent with previous studies from major ocean gyres [61] and the offshore western Mediterranean [62]. In contrast, the PA community was more abundant (observed_ASVs) than the FL counterparts. We should caution that these differences could, in principle, be due to more 16S rRNA genes per genome in the PA bacteria compared with the FL ones [12].

The microbial community structure varied between size fractions. The FL and PA communities were enriched in different taxa and differed significantly (Figure 3b). For example, Gammaproteobacteria, Planctomycetota, Firmicutes, Verucomicrobiota were enriched in the PA, which is consistent with previous studies, such as those in the Atlantic [27], North Pacific Subtropical Gyre [63], Baltic [21] and Mediterranean Seas [11]. In contrast, Marinimicrobia_(SAR406_clade), Actinobacteriota, SAR324_clade (Marine_group_B), Alphaproteobacteria were enriched in the FL. It is possible that taxa enriched in PA or FL perform different ecological functions. These results agree with the findings in previous studies [12,63]. In addition, the change of the community with sampling depth was clear. Cyanobacteria and Bacteroidetes were relatively more abundant in the surface, whereas Gammaproteobacteria, Marinimicrobia and Bdellovibrionota were relatively more abundant in deeper samples. A similar pattern has been documented previously in the Mediterranean stratified and mixed water columns [64].

### 4.2. Different Environmental Factors Affecting the FL and PA Bacterioplankton Communities

Macronutrient profiles showed that from the surface down to the bottom, various environmental factors changed markedly with depth (Figure S1). These results suggest that the water column was stratified [10,62]. Regarding environmental factors influencing FL and PA bacterioplankton composition and abundance, our study revealed that physical variables (e.g., salinity and temperature), nutrient conditions (e.g., nitrate, nitrite, silicate and phosphate) and spatial factors together contributed to the shifts in bacterioplankton communities in the continental shelf and slope regions (Figure 5a,b). However, the main factors driving the FL and PA community structures were fundamentally different. The variation partitioning analysis (VPA) clearly showed the largest contribution to the variation in the particle-associated bacterioplankton was from environmental factors, whereas the largest contribution to the variation in the FL community was spatial factor. It is possible that the PA community may in general be more metabolically plastic or more responsive to local conditions than the FL [12]. As previously reported, in comparison to the FL, bacterioplankton that dwell on particles exhibit a copiotrophic rather than oligotrophic lifestyle, and are better suited to nutrient-rich and variable marine environments rather than the relatively stable oligotrophic seawater environments [12,46].

In this study, temperature and salinity appeared to play a key role in shaping the FL bacterioplankton community (Figure 5c); conversely, phosphate, nitrite and silicate can explain more community variation in the PA (Figure 5d). The results of our study indicate that FL bacteria are more susceptible to environmental changes (in this case temperature and salinity) compared to PA bacteria, because FL cells are more directly exposed to the surrounding water [65,66]. This can be expected as particles, to which PA bacteria are attached, may act as a “buffer” or micro-island, thereby, PA bacteria are impacted by macronutrients determined by the local environment [65]. Nevertheless, the unexplained contributing part to the variation in the bacterioplankton community was more than 50%, suggesting that other environmental factors, which were not measured in this study, are also important drivers of the bacterioplankton community. For example, a previous study indicated that the combination of the matrices of pigments and depth had significant explanatory power on FL community [67]. Consequently, our understanding of the relative importance of all processes governing community assembly remains incomplete.

### 4.3. Stochastic Processes Dominated Bacterioplankton Community Assembly

Because of the limitation of variation partitioning analysis for inferring ecological processes [68], it is important to interrogate further the ecological mechanisms governing the assembly of microbial communities in community ecology [31,38,54]. In the current study, we used two different conceptual frameworks respectively based on neutral mode and null model, which have been considered useful for investigations of the assembly processes of bacterial communities in diverse environments [38,69,70]. Our results revealed that environmental drift (stochastic process) overwhelmed homogeneous selection (deterministic process) in the bacterioplankton community assembly regardless of free-living or particle-associated fractions. It agrees with some recent studies that focused on bacterioplankton in estuarine and coastal environments [25]. The null model based on iCAMP showed that homogeneous selection was more responsible for the assembly and turnover of the FL community, while drift and dispersal limitation contributed more to the community assembly in the PA community (Figure 7). That may be because of a higher habitat homogeneity and hydrologic connectivity in the FL community than in the PA community. Homogeneous selection led to the observed reduction of species richness and more similar community composition under consistent environmental conditions [21,71], which is consistent with the lower species richness and Pielou indices in the FL community than the PA community (Supporting Information Figure S2). It may be because of the effect of different light, oxygen and nutrient conditions in size-fractionated samples (free living and particle associated) on the bacterioplankton or due to biotic interactions (e.g., competition, facilitation, mutualism and predation) [68,72]. Stochastic processes, such as dispersal and drift, are important for controlling the assembly of microbial communities in aquatic environments, including rivers, lakes and marine ecosystems [73], where dispersal is relatively unrestricted. In the current study, drift and dispersal limitation contributed more to the community assembly in the particle-associated fractions. It is possible that hydrologic mixing increases dispersal-related processes and ecological drift in open fluidic systems [74,75].

Since the null model approach could more closely reflect the actual stochastic population dynamics, we also performed a neutral community modeling analysis [31,68]. Our finding suggests that neutrally distributed ASVs contributed a large proportion of community richness and thus that community assembly was predominantly driven by neutral processes (Figure 6). The value of the neutral model parameter R^2^ was slightly higher in the FL community than in the PA community, indicating that the influences of stochastic processes were stronger for the FL. These observations might be explained by greater habitat homogeneity and hydrologic connectivity in the FL than in the PA, as indicated by the difference of the Bray–Curtis distance between the two fractions (Figure 2; Supporting Information Figure S3). Moreover, the result of immigration ratio showed that the PA was more dispersal limited than the FL, which was consistent with the result of null model analysis (Figure 6 and Figure 7a,b). However, the neutral interpretation provided a better fit for the continental slope samples than for the continental shelf samples in the FL, just the reverse in the PA, indicating that the influence of stochastic processes increased in the PA and decreased in the FL from the shelf to the slope. These observations might be explained by the decreased contribution of land-derived particles and more variable hydrology in the environment farther away from the coast at a marginal sea, for example, seawater density and temperature, which were the most important environmental modulators of the balance between stochastic and deterministic assembly processes [29].

These two statistical frameworks provide complementary information and have their advantages and limitations. Many previous studies have pointed out that aquatic microorganisms may frequently alternate between a free-living and a particle-associated stage under environmental stress [76,77]. Whether this alternation occurs for the bacterioplankton communities in the South China Sea remains to be investigated in the future.

### 4.4. Predicted Metabolic Potential in the FL and PA Communities

Understanding the functional profiles of bacterial communities is of great importance because it may shed light on ecosystem processes and community assembly mechanisms. In our present study, functional predictions using PICRUSt2 revealed that the bacteria in the subtropical marginal sea in China were involved in many diverse pathways (Figure 8), most of which were the metabolism of carbohydrates, cofactors, amino acids, terpenoids, polyketides, lipids and biosynthesis of other secondary metabolites. However, some specific metabolic pathways were enriched in the PA community. For example, the PA community significantly enriched in genes mediating antibiotic resistance than in the FL community [78]. Compared to the PA community, proteasome was more abundant in the FL community. Studies have shown that proteasome plays a vital role in mycobacterial and actinobacterial stress response pathways in hostile conditions [79]. How this potential function fits the environment being studied remains unclear currently.

## 5. Conclusions

In this study, bacterioplankton communities at the continental shelf and slope stations in a subtropical marginal sea were analyzed from 45 samples based on 16S rRNA gene high-throughput sequencing. Both bacterioplankton communities and environmental variables exhibited significant spatial variations. More diverse bacteria were found in the FL community compared to the PA community. Moreover, some members of Marinimicrobia_(SAR406_clade), Actinobacteriota, SAR324_clade(Marine_group_B), Alphaproteobacteria and Cyanobacteria were more abundantly represented in the FL commuities, whereas *Gammaproteobacteria*, *Planctomycetota*, *Firmicutes*, *Verucomicrobiota* and *Desulfobacterota* were enriched in the PA community. Moreover, our results demonstrated that salinity and water temperature were the key factors modulating turnover in the FL bacterioplankton, whereas nitrite, silicate and phosphate were the key factors modulating turnover in the PA counterparts. Finally, our findings suggested that deterministic and stochastic processes simultaneously affected the assembly of bacterioplankton communities, in which the stochastic processes exerted stronger influences on the FL bacterioplankton community, whereas deterministic processes had stronger effects on the PA community. Meanwhile, we found that homogeneous selection was more responsible for the assembly and turnover of the FL community, while drift and dispersal limitation contributed more to the community assembly in the PA community. Importantly, the major contributor to HoS in the PA community was Gammaproteobacteria:Others, whereas in the FL community was Cyanobacteria:Bin6. Addtionally, the major contributors to drift and dispersal limitation in both the PA community and FL community were Gammaproteobacteria:Bin91 and Gammaproteobacteria:Others, respectively. In addition, the predicted metagenomic analysis (PICRUSt2) indicated that the potential metabolism of carbohydrates, cofactors, amino acids, terpenoids, polyketides, lipids, biosynthesis of other secondary metabolites and antibiotic resistance were also markedly more enriched in the PA than the FL community. The findings of differential influencing factors and assembly processes between the FL and PA bacterioplankton communities provide a foundation for further studies to understand the dynamics and ecological niches of these communities in the continental shelf and slope regions.

## Figures and Tables

**Table 1:** Criteria to quantify the importance of ecological process accounting for the turnover between communities following Ning et al. (2020).

Process.	Definition	βNRI	RC
Homogeneous selection	Consistent selective pressure resulting from consistent environmental conditions is the primary cause of compositional turnover between a pair of local communities	<−1.96	
Heterogeneous selection	Divergent selective pressure resulting from divergent environmental conditions is the primary cause of compositional turnover between a pair of local communities	>+1.96	
Homogenizing dispersal	High dispersal rates between a pair of local communities is the primary cause of compositional turnover between a pair of local communities	<|1.96|	<−0.95
Dispersal limitation	Low dispersal rates between a pair of local communities, acting in concert with ecological drift, are the primary cause of compositional turnover between a pair of local communities	<|1.96|	>+0.95
Drift (and others)	Neither selection nor dispersal is the dominant process driving compositional turnover between a pair of local communities	<|1.96|	<|0.95|

## Data Availability

All data generated or analysed during this study are included in this article and its supplementary material files. Further enquiries can be directed to the corresponding author (ED).

## Supplementary Materials

The following are available online at https://www.mdpi.com/article/10.3390/microorganisms9122445/s1, Figure S1. An overview of environmental and prokaryotic data at each depth in both the C6 and C9 sampled stations, Figure S2. Distribution of average alpha-diversity at each depth, size-fraction for both the C6 and C9 sampled stations, Figure S3. nMDS ordinations representing the Bray–Curtis distance between bacteiroplankton communities, Figure S4. Relative abundance of top nine bacterial taxa in the Phylum/class level, Figure S5. Relative abundance of top nine bacterial taxa at the family level, Figure S6. Ecological processes controlling major phylogenetic bins, Table S1. Permutational multivariate analysis of variance (PERMANOVA) examining the effects of the factors station, depth and size-fraction on bacterioplankton community structure, Table S2. Group information of all samples, Table S3. Results of two-way ANOVAs with factors for habitat and depth interaction for the alpha diversity indices of bacterioplankton communities, Table S4. Summary of the coefficient of determination (R^2^) from the Permutational multivariate analysis of variance (PERMANOVA) examining the effects of the factors station and depth for each of the two size-fractions, Table S5. ANOSIM (analysis of similarity) statistics of bacterioplankton community composition between samples grouped following Figure 2, Table S6 Fit of the neutral model for bacterioplankton community, Table S7 Summary of the contribution of the ecological processes that determine community assembly of bacterioplankton Continental Shelf-Slope Region in northern South China Sea.

## References

[1] Freimann R., Bürgmann H., Findlay S.E., Robinson C.T. (2013). Bacterial structures and ecosystem functions in glaciated floodplains: Contemporary states and potential future shifts. ISME J..

[2] Lopes Dos Santos A., Gourvil P., Tragin M., Noel M.H., Decelle J., Romac S., Vaulot D. (2017). Diversity and oceanic distribution of prasinophytes clade VII, the dominant group of green algae in oceanic waters. ISME J..

[3] Gutknecht J.L.M., Field C.B., Balser T.C. (2012). Microbial communities and their responses to simulated global change fluctuate greatly over multiple years. Glob. Chang. Biol..

[4] Azam F., Malfatti F. (2007). Microbial structuring of marine ecosystems. Nat. Rev. Genet..

[5] Guidi L., Chaffron S., Bittner L., Eveillard D., Larhlimi A., Roux S., Darzi Y., Audic S., Berline L., Brum J.R. (2016). Plankton networks driving carbon export in the oligotrophic ocean. Nature.

[6] Letscher R.T., Knapp A.N., James A.K., Carlson C.A., Santoro A., Hansell D. (2015). Microbial community composition and nitrogen availability influence DOC remineralization in the South Pacific Gyre. Mar. Chem..

[7] Dang H., Lovell C.R. (2016). Microbial Surface Colonization and Biofilm Development in Marine Environments. Microbiol. Mol. Biol. Rev..

[8] Azam F., Long R.A. (2001). Sea snow microcosms. Nature.

[9] Aristegui J., Gasol J.M., Duarte C.M., Herndl G.J. (2009). Microbial oceanography of the dark ocean’s pelagic realm. Limnol. Oceanogr..

[10] Karl D.M. (2007). Microbial oceanography: Paradigms, processes and promise. Nat. Rev. Genet..

[11] Mestre M., Borrull E., Sala M.M., Gasol J.M. (2017). Patterns of bacterial diversity in the marine planktonic particulate matter continuum. ISME J..

[12] Luo H., Moran M.A. (2015). How do divergent ecological strategies emerge among marine bacterioplankton lineages?. Trends Microbiol..

[13] Bižić-Ionescu M., Amann R., Grossart H.-P. (2014). Massive Regime Shifts and High Activity of Heterotrophic Bacteria in an Ice-Covered Lake. PLoS ONE.

[14] Tang X., Chao J., Gong Y., Wang Y., Wilhelm S., Gao G. (2017). Spatiotemporal dynamics of bacterial community composition in large shallow eutrophic Lake Taihu: High overlap between free-living and particle-attached assemblages. Limnol. Oceanogr..

[15] Xu H., Zhao D., Huang R., Cao X., Zeng J., Yu Z., Hooker K.V., Hambright K.D., Wu Q.L. (2018). Contrasting Network Features between Free-Living and Particle-Attached Bacterial Communities in Taihu Lake. Microb. Ecol..

[16] Savio D., Sinclair L., Ijaz U.Z., Parajka J., Reischer G., Stadler P., Blaschke A.P., Blöschl G., Mach R., Kirschner A. (2015). Bacterial diversity along a 2600 km river continuum. Environ. Microbiol..

[17] Henson M.W., Hanssen J., Spooner G., Fleming P., Pukonen M., Stahr F., Thrash J.C. (2018). Nutrient dynamics and stream order influence microbial community patterns along a 2914 kilometer transect of the Mississippi River. Limnol. Oceanogr..

[18] Yoshimura K., York J., Biddle J.F. (2018). Impacts of Salinity and Oxygen on Particle-Associated Microbial Communities in the Broadkill River, Lewes DE. Front. Mar. Sci..

[19] Smith M.W., Allen L.Z., Allen A.E., Herfort L., Simon H.M. (2013). Contrasting genomic properties of free-living and particle-attached microbial assemblages within a coastal ecosystem. Front. Microbiol..

[20] Bižić-Ionescu M., Zeder M., Ionescu D., Orlic S., Fuchs B., Grossart H.-P., Amann R. (2014). Comparison of bacterial communities on limnic versus coastal marine particles reveals profound differences in colonization. Environ. Microbiol..

[21] Rieck A., Herlemann D., Jürgens K., Grossart H.-P. (2015). Particle-Associated Differ from Free-Living Bacteria in Surface Waters of the Baltic Sea. Front. Microbiol..

[22] Doherty M., Yager P., Moran M.A., Coles V.J., Fortunato C.S., Krusche A., Medeiros P.M., Payet J.P., Richey J.E., Satinsky B. (2017). Bacterial Biogeography across the Amazon River-Ocean Continuum. Front. Microbiol..

[23] Kieft B., Li Z., Bryson S., Crump B.C., Hettich R., Pan C., Mayali X., Mueller R.S. (2018). Microbial Community Structure–Function Relationships in Yaquina Bay Estuary Reveal Spatially Distinct Carbon and Nitrogen Cycling Capacities. Front. Microbiol..

[24] Xie W., Luo H., Murugapiran S.K., Dodsworth J.A., Chen S., Sun Y., Hedlund B.P., Wang P., Fang H., Deng M. (2018). Localized high abundance of Marine Group II archaea in the subtropical Pearl River Estuary: Implications for their niche adaptation. Environ. Microbiol..

[25] Wang Y., Pan J., Yang J., Zhou Z., Pan Y., Li M. (2020). Patterns and processes of free-living and particle-associated bacterioplankton and archaeaplankton communities in a subtropical river-bay system in South China. Limnol. Oceanogr..

[26] Salazar G., Cornejo-Castillo F.M., Benítez-Barrios V., Fraile-Nuez E., Alvarez-Salgado X.A., Duarte C.M., Gasol J.M., Acinas S.G. (2016). Global diversity and biogeography of deep-sea pelagic prokaryotes. ISME J..

[27] Milici M., Deng Z.L., Tomasch J., Decelle J., Wos-Oxley M.L., Wang H., Jauregui R., Plumeier I., Giebel H.A., Badewien T.H. (2016). Co-occurrence Analysis of Microbial Taxa in the Atlantic Ocean Reveals High Connectivity in the Free-Living Bacterioplankton. Front. Microbiol..

[28] Suter E.A., Pachiadaki M., Taylor G.T., Astor Y., Edgcomb V.P. (2018). Free-living chemoautotrophic and particle-attached heterotrophic prokaryotes dominate microbial assemblages along a pelagic redox gradient. Environ. Microbiol..

[29] Allen R., Hoffmann L.J., Larcombe M.J., Louisson Z., Summerfield T.C. (2020). Homogeneous environmental selection dominates microbial community assembly in the oligotrophic South Pacific Gyre. Mol. Ecol..

[30] Liu J., Meng Z., Liu X., Zhang X.-H. (2019). Microbial assembly, interaction, functioning, activity and diversification: A review derived from community compositional data. Mar. Life Sci. Technol..

[31] Zhou J., Ning D. (2017). Stochastic Community Assembly: Does It Matter in Microbial Ecology?. Microbiol. Mol. Biol. Rev..

[32] Nemergut D.R., Schmidt S.K., Fukami T., O’Neill S.P., Bilinski T.M., Stanish L.F., Knelman J.E., Darcy J.L., Lynch R.C., Wickey P. (2013). Patterns and Processes of Microbial Community Assembly. Microbiol. Mol. Biol. Rev..

[33] Chase J.M., Leibold M.A. (2004). Ecological Niches: Linking Classical and Contemporary Approaches.

[34] Jetschke G. (2002). The Unified Neutral Theory of Biodiversity and Biogeography. Ecology.

[35] Vellend M. (2010). Conceptual Synthesis in Community Ecology. Q. Rev. Biol..

[36] Vellend M., Srivastava D.S., Anderson K.M., Brown C.D., Jankowski J.E., Kleynhans E.J., Kraft N., Letaw A.D., Macdonald A.A.M., Maclean J.E. (2014). Assessing the relative importance of neutral stochasticity in ecological communities. Oikos.

[37] Vellend M. (2016). The Theory of Ecological Communities.

[38] Ning D., Yuan M., Wu L., Zhang Y., Guo X., Zhou X., Yang Y., Arkin A.P., Firestone M.K., Zhou J. (2020). A quantitative framework reveals ecological drivers of grassland microbial community assembly in response to warming. Nat. Commun..

[39] Stegen J., Lin X., Fredrickson J.K., Chen X., Kennedy D., Murray C.J., Rockhold M.L., Konopka A. (2013). Quantifying community assembly processes and identifying features that impose them. ISME J..

[40] Stegen J., Lin X., Konopka A.E., Fredrickson J.K. (2012). Stochastic and deterministic assembly processes in subsurface microbial communities. ISME J..

[41] Wu W., Lu H.-P., Sastri A., Yeh Y.-C., Gong G.-C., Chou W.-C., Hsieh C.-H. (2017). Contrasting the relative importance of species sorting and dispersal limitation in shaping marine bacterial versus protist communities. ISME J..

[42] Mo Y., Zhang W., Yang J., Lin Y., Yu Z., Lin S. (2018). Biogeographic patterns of abundant and rare bacterioplankton in three subtropical bays resulting from selective and neutral processes. ISME J..

[43] Wang K., Yan H., Peng X., Hu H., Zhang H., Hou D., Chen W., Qian P., Liu J., Cai J. (2020). Community assembly of bacteria and archaea in coastal waters governed by contrasting mechanisms: A seasonal perspective. Mol. Ecol..

[44] Ren L., Song X., He D., Wang J., Tan M., Xia X., Li G., Tan Y., Wu Q.L. (2019). Bacterioplankton Metacommunity Processes across Thermal Gradients: Weaker Species Sorting but Stronger Niche Segregation in Summer than in Winter in a Subtropical Bay. Appl. Environ. Microbiol..

[45] Mollenhauer G., Schneider R., Müller P.J., Spiess V., Wefer G. (2002). Glacial/interglacial variablity in the Benguela upwelling system: Spatial distribution and budgets of organic carbon accumulation. Glob. Biogeochem. Cycles.

[46] Satinsky B., Crump B., Smith C.B., Sharma S., Zielinski B.L., Doherty M., Meng J., Sun S., Medeiros P.M., Paul J.H. (2014). Microspatial gene expression patterns in the Amazon River Plume. Proc. Natl. Acad. Sci. USA.

[47] Simon H.M., Smith M.W., Herfort L. (2014). Metagenomic insights into particles and their associated microbiota in a coastal margin ecosystem. Front. Microbiol..

[48] Yuan J., Li M., Lin S. (2015). An Improved DNA Extraction Method for Efficient and Quantitative Recovery of Phytoplankton Diversity in Natural Assemblages. PLoS ONE.

[49] Caporaso J.G., Lauber C.L., Walters W.A., Berg-Lyons D., Lozupone C.A., Turnbaugh P.J., Fierer N., Knight R. (2011). Global patterns of 16S rRNA diversity at a depth of millions of sequences per sample. Proc. Natl. Acad. Sci. USA.

[50] Bolyen E., Rideout J.R., Dillon M.R., Bokulich N.A., Abnet C.C., Al-Ghalith G.A., Alexander H., Alm E.J., Arumugam M., Asnicar F. (2019). Reproducible, interactive, scalable and extensible microbiome data science using QIIME 2. Nat. Biotechnol..

[51] Callahan B.J., Mcmurdie P.J., Rosen M.J., Han A.W., Johnson A.J., Holmes S.P. (2016). DADA2: High-resolution sample inference from Illumina amplicon data. Nat. Methods.

[52] Price M.N., Dehal P.S., Arkin A.P. (2009). FastTree: Computing Large Minimum Evolution Trees with Profiles instead of a Distance Matrix. Mol. Biol. Evol..

[53] Caicedo H.H., Hashimoto D.A., Caicedo J.C., Pentland A., Pisano G.P. (2020). Overcoming barriers to early disease intervention. Nat. Biotechnol..

[54] Sloan W.T., Lunn M., Woodcock S., Head I.M., Nee S., Curtis T.P. (2006). Quantifying the roles of immigration and chance in shaping prokaryote community structure. Environ. Microbiol..

[55] Logares R., Lindström E., Langenheder S., Logue J.B., Paterson H., Laybourn-Parry J., Rengefors K., Tranvik L.J., Bertilsson S. (2012). Biogeography of bacterial communities exposed to progressive long-term environmental change. ISME J..

[56] Liao J., Cao X., Wang J., Zhao L., Sun J., Jiang D., Huang Y. (2017). Similar community assembly mechanisms underlie similar biogeography of rare and abundant bacteria in lakes on Yungui Plateau, China. Limnol. Oceanogr..

[57] Hammer Ø., Harper D.A., Ryan P.D. (2001). PAST: Paleontological Statistics Software Package for Education and Data Analysis. Palaeontol. Electron..

[58] Parks D.H., Tyson G.W., Hugenholtz P., Beiko R.G. (2014). STAMP: Statistical analysis of taxonomic and functional profiles. Bioinformatics.

[59] Lauro F.M., McDougald D., Thomas T., Williams T.J., Egan S., Rice S., DeMaere M., Ting L., Ertan H., Johnson J. (2009). The genomic basis of trophic strategy in marine bacteria. Proc. Natl. Acad. Sci. USA.

[60] Mao X., Chen J., van Oosterhout C., Zhang H., Liu G., Zhuang Y., Mock T. (2021). Diversity, prevalence, and expression of cyanase genes (cynS) in planktonic marine microorganisms. ISME J..

[61] Ghiglione J.F., Mevel G., Pujo-Pay M., Mousseau L., Lebaron P., Goutx M. (2007). Diel and Seasonal Variations in Abundance, Activity, and Community Structure of Particle-Attached and Free-Living Bacteria in NW Mediterranean Sea. Microb. Ecol..

[62] Mestre M., González C.R., Logares R., Duarte C.M., Gasol J.M., Sala M.M. (2018). Sinking particles promote vertical connectivity in the ocean microbiome. Proc. Natl. Acad. Sci. USA.

[63] Fontanez K.M., Eppley J.M., Samo T., Karl D., Delong E.F. (2015). Microbial community structure and function on sinking particles in the North Pacific Subtropical Gyre. Front. Microbiol..

[64] Haro-Moreno J.M., Lopez-Perez M., de la Torre J.R., Picazo A., Camacho A., Rodriguez-Valera F. (2018). Fine metagenomic profile of the Mediterranean stratified and mixed water columns revealed by assembly and recruitment. Microbiome.

[65] Yung C.-M., Ward C.S., Davis K.M., Johnson Z.I., Hunt D.E. (2016). Insensitivity of Diverse and Temporally Variable Particle-Associated Microbial Communities to Bulk Seawater Environmental Parameters. Appl. Environ. Microbiol..

[66] Bachmann J., Heimbach T., Hassenrück C., Kopprio G., Iversen M.H., Grossart H.P., Gärdes A. (2018). Environmental Drivers of Free-Living vs. Particle-Attached Bacterial Community Composition in the Mauritania Upwelling System. Front. Microbiol..

[67] Roth Rosenberg D., Haber M., Goldford J., Lalzar M., Aharonovich D., Al-Ashhab A., Lehahn Y., Segre D., Steindler L., Sher D. (2021). Particle-associated and free-living bacterial communities in an oligotrophic sea are affected by different environmental factors. Environ. Microbiol..

[68] Langenheder S., Lindström E.S. (2019). Factors influencing aquatic and terrestrial bacterial community assembly. Environ. Microbiol. Rep..

[69] Roguet A., Laigle G.S., Therial C., Bressy A., Soulignac F., Catherine A., Lacroix G., Jardillier L., Bonhomme C., Lerch T.Z. (2015). Neutral community model explains the bacterial community assembly in freshwater lakes. FEMS Microbiol. Ecol..

[70] Burns A., Stephens W.Z., Stagaman K., Wong S., Rawls J., Guillemin K., Bohannan B.J. (2016). Contribution of neutral processes to the assembly of gut microbial communities in the zebrafish over host development. ISME J..

[71] Fuhrman J.A., Cram J., Needham D.M. (2015). Marine microbial community dynamics and their ecological interpretation. Nat. Rev. Genet..

[72] Bižić-Ionescu M., Ionescu D., Grossart H.-P. (2018). Organic Particles: Heterogeneous Hubs for Microbial Interactions in Aquatic Ecosystems. Front. Microbiol..

[73] Zhou J., Deng Y., Zhang P., Xue K., Liang Y., Van Nostrand J.D., Yang Y., He Z., Wu L., Stahl D.A. (2014). Stochasticity, succession, and environmental perturbations in a fluidic ecosystem. Proc. Natl. Acad. Sci. USA.

[74] Meyerhof M.S., Wilson J.M., Dawson M.N., Beman J.M. (2016). Microbial community diversity, structure and assembly across oxygen gradients in meromictic marine lakes, Palau. Environ. Microbiol..

[75] Bracken L., Wainwright J., Ali G., Tetzlaff D., Smith M., Reaney S.M., Roy A. (2013). Concepts of hydrological connectivity: Research approaches, pathways and future agendas. Earth-Sci. Rev..

[76] Grossart H.-P., Tang K.W. (2010). www.aquaticmicrobial.net. Commun. Integr. Biol..

[77] Liu M., Liu L., Chen H., Yu Z., Yang J.R., Xue Y., Huang B., Yang J. (2019). Community dynamics of free-living and particle-attached bacteria following a reservoir Microcystis bloom. Sci. Total Environ..

[78] Ganesh S., Parris D.J., Delong E.F., Stewart F.J. (2013). Metagenomic analysis of size-fractionated picoplankton in a marine oxygen minimum zone. ISME J..

[79] Von Rosen T., Keller L.M.L., Weber-Ban E. (2021). Survival in Hostile Conditions: Pupylation and the Proteasome in Actinobacterial Stress Response Pathways. Front. Mol. Biosci..

