# Supplementary material for: Diversity Distribution, Driving Factors and Assembly Mechanisms of Free-Living and Particle-Associated Bacterial Communities at a Subtropical Marginal Sea
Source: Microorganisms. 2021 Nov 27;9(12):2445. doi: 10.3390/microorganisms9122445 (PMC8704526; doi:10.3390/microorganisms9122445)
Supplement: Supplementary file 1 [file microorganisms-09-02445-s001.zip › microorganisms-1467569-supplementary/Supplementary Materials-revisions.pdf]

Supplementary Information

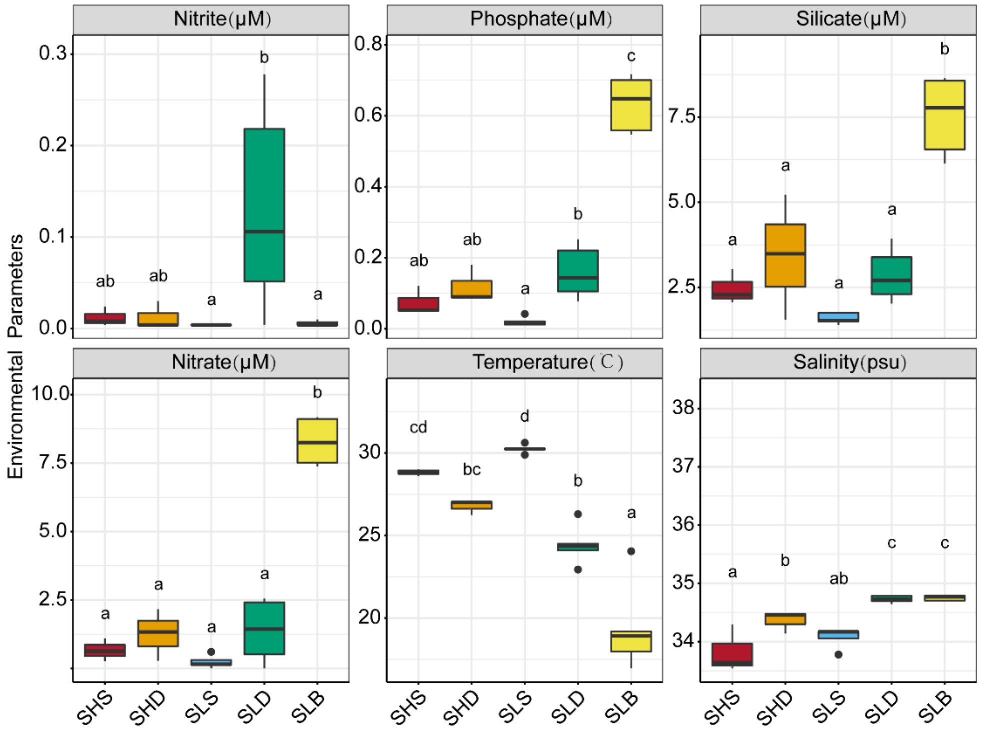

Fig. S1. An overview of environmental data at each depth in both the C6 and C9 sampled stations. The boxplots are constructed with the first and third quartiles of the distribution of values, and the medians. The lines extending from the boxes (whiskers) indicate the variability outside the first and third quartiles. Data are expressed as mean ± sd. Different alphabets above data bars indicate significantly different values ($P < 0.05$, ANOVA, Tukey's HSD).

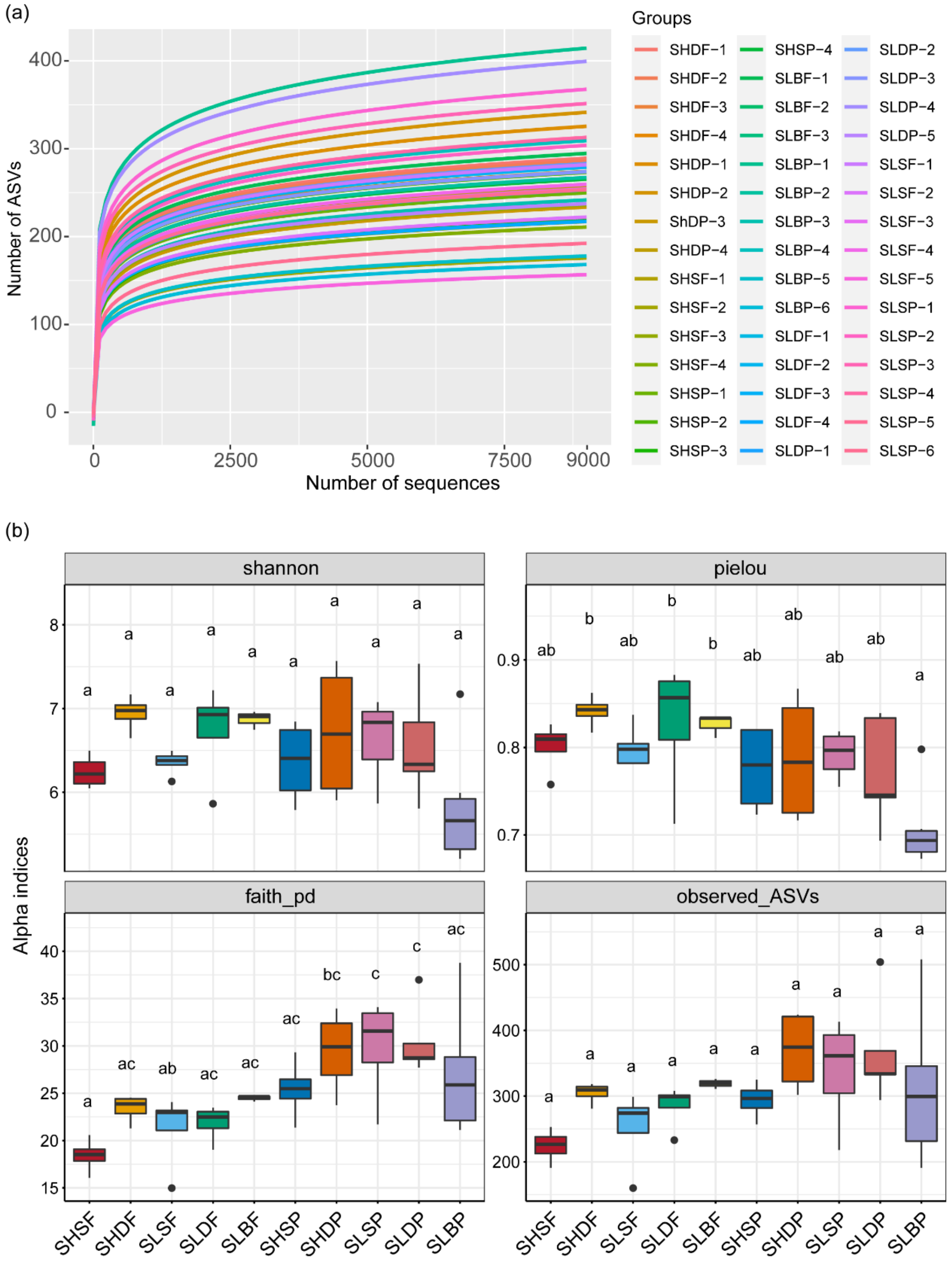

Fig. S2. (a) Rarefaction curves of detected bacterial species of all the samples; (b) Distribution of average alpha-diversity at each group. The boxplots are constructed with the first and third quartiles of the distribution of values, and the medians. The lines extending from the boxes (whiskers) indicate the variability outside the first and third quartiles. Data are expressed as mean ±sd. Different alphabets above data bars indicate significantly different values ($P < 0.05$, ANOVA, Tukey's HSD).

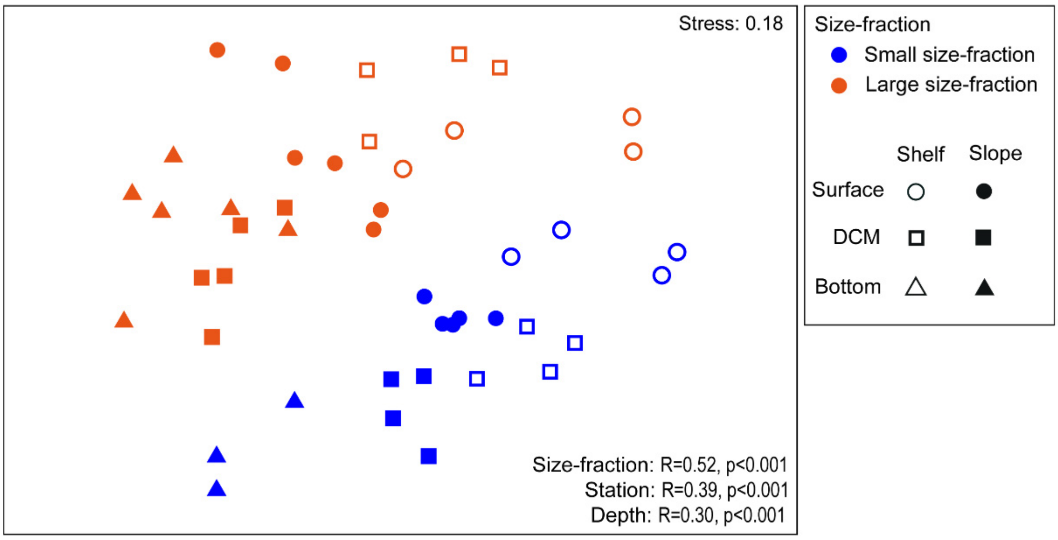

Fig. S3. nMDS ordinations representing the Bray-Curtis distance between bacterioplankton communities. Anosim analysis of the effects of station, depth and size-fraction on bacterioplankton community similarity.

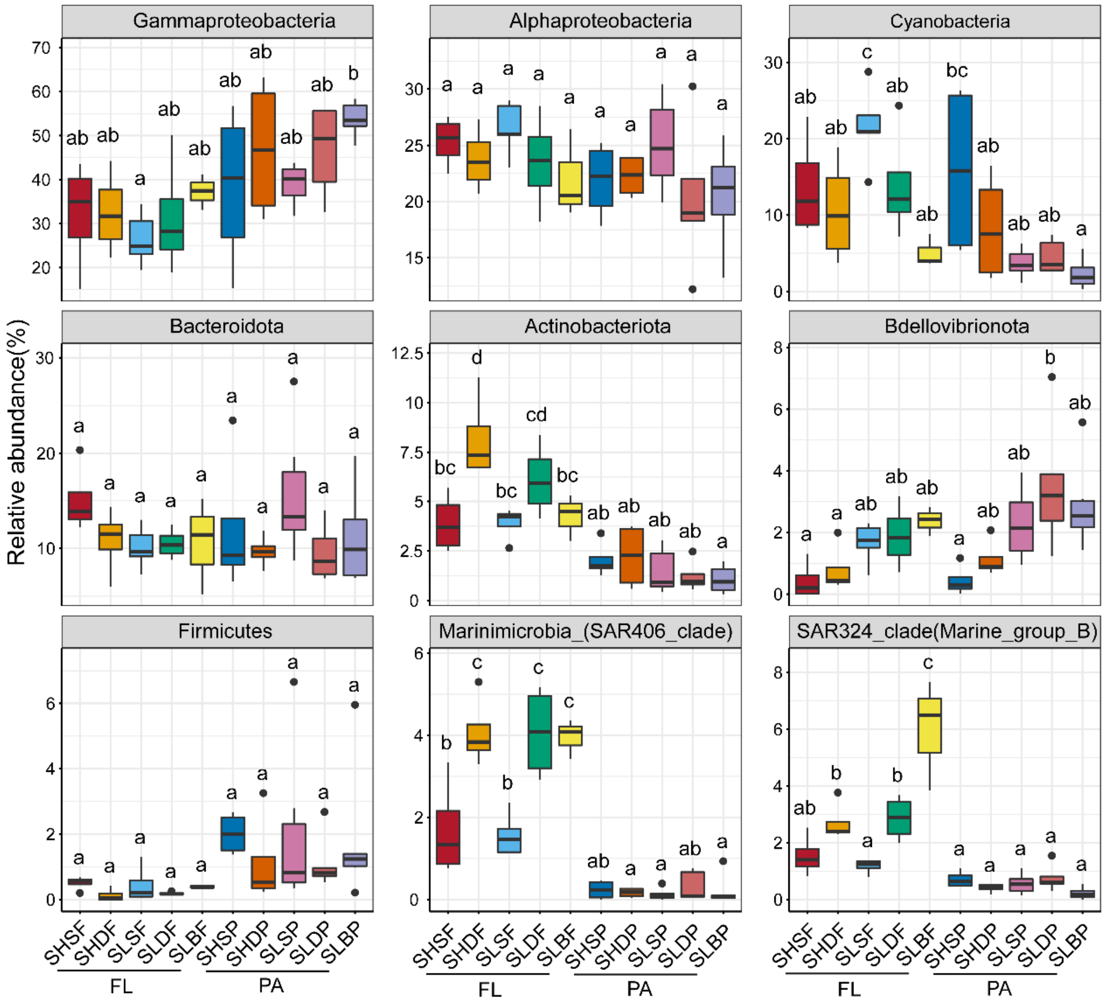

Fig. S4. Relative abundance of top nine bacterial taxa at the Phylum/class level. The median values are shown with horizontal black wide lines. Boxplots were constructed with the upper and lower lines corresponding to the first and third quartile of the distribution of values, and the lower and upper whiskers represent the minimum and maximum of all data, respectively. Data are expressed as mean ± sd. Different alphabets above data bars indicate significantly different values ($P < 0.05$, ANOVA, Tukey's HSD).

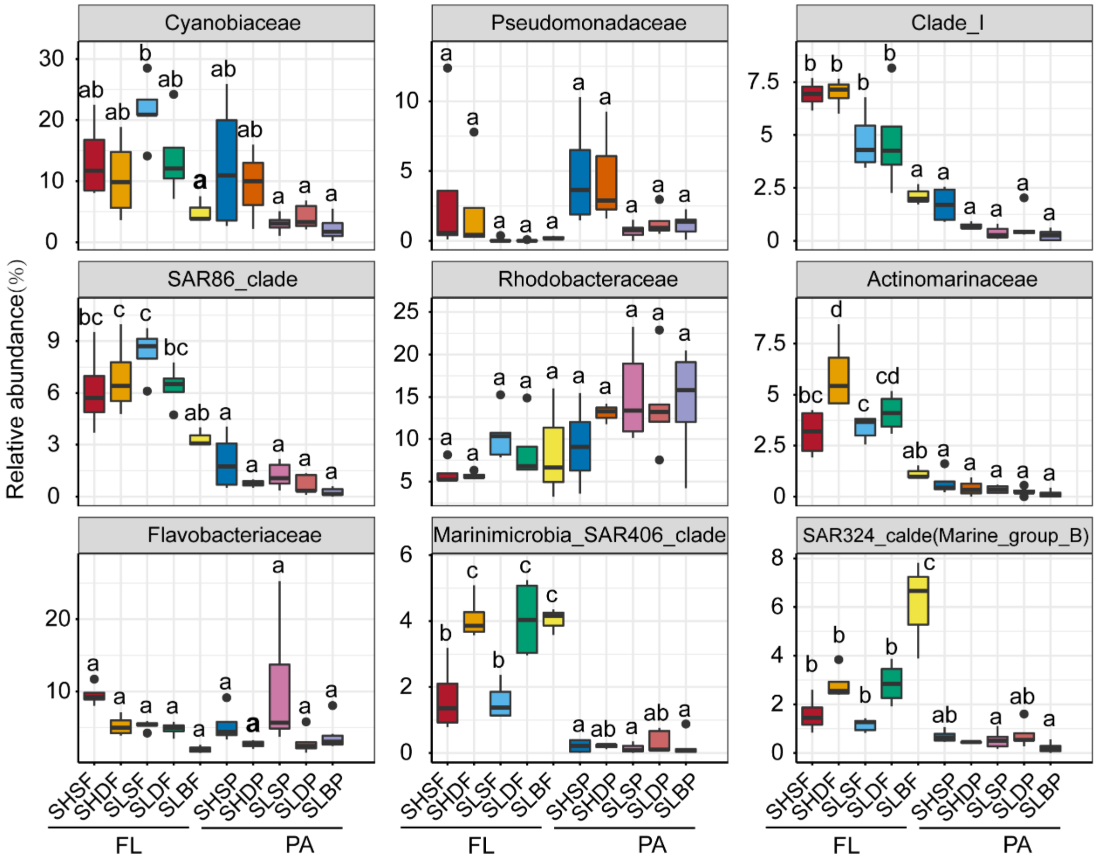

Fig. S5. Relative abundance of top nine bacterial taxa at the family level. The median values are shown with horizontal black thick lines. Boxplots were constructed with the top and bottom corresponding to the first and third quartile of the distribution of values, and the lower and upper whiskers represent the minimum and maximum of all data, respectively. Data are expressed as mean ± sd. Different alphabets above data bars indicate significantly different values ($P < 0.05$, ANOVA, Tukey's HSD).

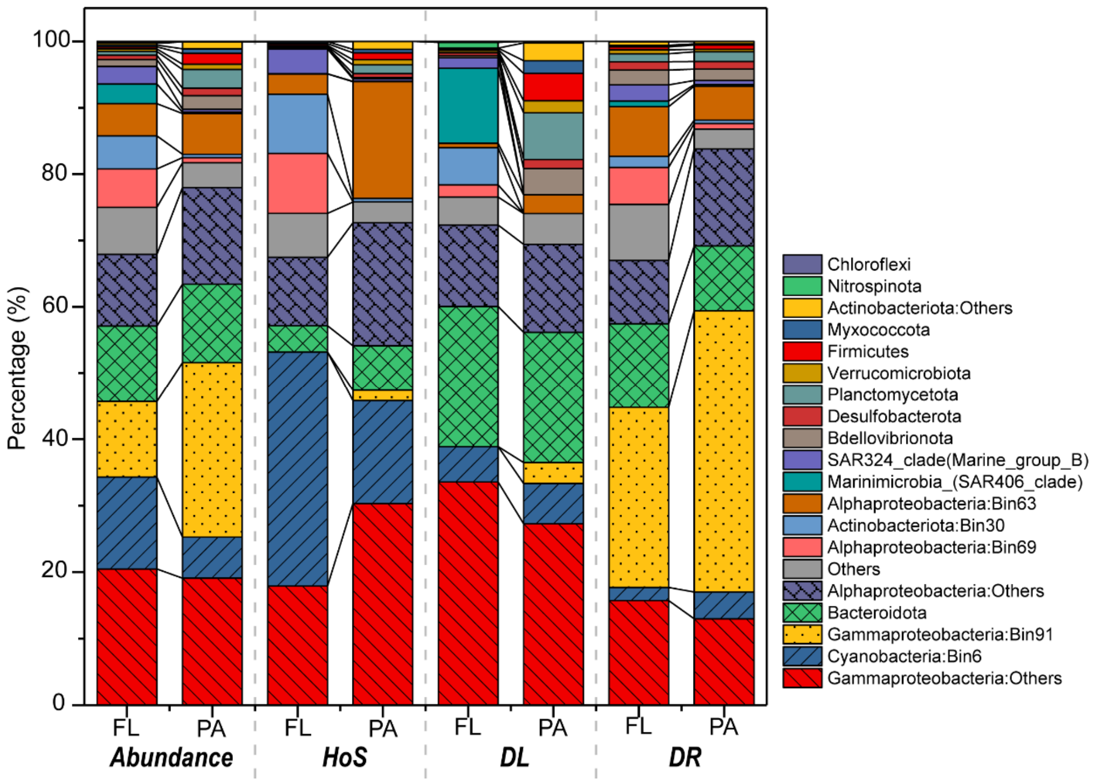

Fig. S6. Ecological processes controlling major phylogenetic bins. Relative abundances of different phyla and their relative contributions to homogeneous selection (HoS), dispersal limitation(DL) and drift (DR) under free-living (FL) and particle-associated (PA). Top 5 abundant bins were particularly highlighted.

Table S1. Permutational multivariate analysis of variance (PERMANOVA) examining the effects of the factors station, depth and size-fraction on bacterioplankton community structure. Key to abbreviations and column headings: D.f, degrees of freedom; SS, sum of squares; MS, mean square; F, F ratio; R^2, coefficient of determination; P, p-value. *** = p<0.001.

	D.f.	SS	MS	F	R^2	p-value
**Size-fraction**	1	2.18	2.18	9.95	0.19	***
**Station**	1	1.67	1.67	7.24	0.14	***
**Depth**	2	0.80	0.80	3.19	0.069	***
**Residuals**	41	30.21				
**Total**	45	34.86				

Table S2. Group information of all samples. Samples grouped by two size fractions (free-living and particle-associated), different stations (Slope and Shelf), and depth (Surface, DCM and Bottom). Site names: SL = Slope, SH = Shelf, S = Surface, D = DCM, B=Bottom, F = free-living, and P = particle-associated.

**Groups**	**Replicate**	**Size-fraction**		**By Depth**			**By Station**	
		PA(P)	FL(F)	Surface(S)	DCM(D)	Bottom(B)	Shelf(SH)	Slope(SL)
**SHSF**	4		√	√			√	
**SHDF**	4		√		√		√	
**SLSF**	5		√	√				√
**SLDF**	4		√		√			√
**SLBF**	3		√			√		√
**SHSP**	4	√		√			√	
**SHDP**	4	√			√		√	
**SLSP**	6	√		√				√
**SLDP**	5	√			√			√
**SLBP**	6	√				√		√

Table S3. Results of two-way ANOVAs with factors for habitat and depth interaction for the alpha diversity indices of bacterioplankton communities.

Indicators	Factors	F	p-*value*
**shannon**	station	4.302	0.0393
	depth	19.393	1.93e-08
	station:depth	4.649	0.0106
**pielou**	station	12.173	0.000593
	depth	26.039	8.38e-11
	station:depth	1.923	0.148723
**Faith_pd**	station	10.971	0.001094
	depth	5.317	0.005606
	station:depth	8.927	0.000192
**Observed_ASVs**	station	2.682	0.10301
	depth	10.490	4.6e-05
	station:depth	4.989	0.00767

Table S4. Summary of the coefficient of determination (R^2) from the Permutational multivariate analysis of variance (PERMANOVA) examining the effects of the factors station and depth for each of the two size-fractions. ***= p<0.001; **= p<0.01.

	By Depth		By Station	
	R^2	p-value	R^2	p-value
FL	0.174	***	0.226	***
PA	0.098	**	0.185	***

Table S5. ANOSIM (analysis of similarity) statistics of bacterioplankton community composition between samples grouped following Table S2.

Groups	Sample statistic R	No.of Monte carlo permutations with Scores ≥R	P Value
FLvs.PA	0.718	999	0.001
SHSF vs.SHDF	0.490	999	0.051
SHSF vs.SLSF	0.931	999	0.01
SHSF vs.SLDF	1	999	0.031
SHSF vs.SLBF	1	999	0.03
SHSF vs.SHSP	0.260	999	0.161
SHSF vs.SHDP	0.667	999	0.031
SHSF vs.SLSP	0.869	999	0.004
SHSF vs.SLDP	1	999	0.01
SHSF vs.SLBP	1	999	0.005
SHDF vs.SLSF	0.9625	999	0.013
SHDF vs.SLDF	0.667	999	0.028
SHDF vs.SLBF	1	999	0.027
SHDF vs.SHSP	0.646	999	0.025
SHDF vs.SHDP	0.698	999	0.032
SHDF vs.SLSP	0.921	999	0.007
SHDF vs.SLDP	0.988	999	0.008
SHDF vs.SLBP	1	999	0.01
SLSF vs.SLDF	0.806	999	0.009
SLSF vs.SLBF	1	999	0.016
SLSF vs.SHSP	0.875	999	0.007
SLSF vs.SHDP	0.95	999	0.007
SLSF vs.SLSP	0.773	999	0.001
SLSF vs.SLDP	0.956	999	0.012
SLSF vs.SLBP	0.989	999	0.002
SLDF vs.SLBF	1	999	0.032
SLDF vs.SHSP	0.854	999	0.031
SLDF vs.SHDP	0.865	999	0.031
SLDF vs.SLSP	0.869	999	0.013
SLDF vs.SLDP	0.819	999	0.009
SLDF vs.SLBP	0.905	999	0.007
SLBF vs.SHSP	0.741	999	0.059
SLBF vs.SHDP	0.963	999	0.039
SLBF vs.SLSP	0.833	999	0.011
SLBF vs.SLDP	0.733	999	0.016
SLBF vs.SLBP	0.926	999	0.01

SHSP vs.SHDP	0.021	999	0.344
SHSP vs.SLSP	0.548	999	0.009
SHSP vs.SLDP	0.463	999	0.02
SHSP vs.SLBP	0.536	999	0.015
SHDP vs.SLSP	0.623	999	0.007
SHDP vs.SLDP	0.394	999	0.029
SHDP vs.SLBP	0.5	999	0.017
SLSP vs.SLDP	0.403	999	0.016
SLSP vs.SLBP	0.431	999	0.005
SLDP vs.SLBP	0.12	999	0.118

The ANOSIM statistic compares the mean of ranked dissimilarities between groups to the mean of ranked dissimilarities within groups. An R-value close to "1" suggests dissimilarity between groups while an R-value near "0" suggests an even distribution of high and low ranks within and between groups, respectively.

Table S6 Fit of the neutral model for bacterioplankton community. R^2 indicates the fit to the neutral model. Group names: SL = Slope, SH = Shelf, S = Surface, D = DCM, B=Bottom, F = free-living, and P = particle-associated. The R^2 value represents the goodness of fit to the NCM, and the m value represents the migration rate of the species.

**Groups**	**FL**		**PA**	
	**R^2**	**m**	**R^2**	**m**
**SHS**	0.649	0.034	0.564	0.033
**SHD**	0.576	0.052	0.517	0.038
**SLS**	0.655	0.070	0.518	0.033
**SLD**	0.601	0.047	0.560	0.040
**SLB**	0.543	0.048	0.548	0.043

Table S7 Summary of the contribution of the ecological processes that determine community assembly of bacterioplankton Continental Shelf-Slope Region at a subtropical marginal sea . The percentages are given the relative contribution of each process to the community turnover. Group names: SL = Slope, SH = Shelf, S = Surface, D = DCM, B=Bottom, F = free-living, and P = particle-associated. Relative importance of process estimated by iCAMP: Heterogeneous selection (HeS), Homogeneous selection (HoS), Homogenizing dispersal (HD), Dispersal limitation (DL), Drift (DR).

Size-fraction	Groups	HeS (%)	HoS (%)	DL (%)	HD (%)	DR (%)	Stochasticity (%)
FL	SHSF	0.33	29.12	17.47	6.05	47.03	70.76
	SHDF	0.29	30.69	17.90	5.74	45.39	68.97
	SLSF	0.42	39.95	3.68	4.30	51.65	59.97
	SLDF	0.12	37.47	8.46	2.41	51.54	62.29
	SLBF	1.09	30.83	15.87	2.36	49.85	68.14
PA	SHSP	0.08	14.87	25.90	0.81	58.34	85.12
	SHDP	0.32	15.67	22.01	3.82	58.18	84.01
	SLSP	0.42	13.29	27.81	4.04	54.45	86.04
	SLDP	0.68	11.36	20.18	3.36	64.42	88.13
	SLBP	0.08	12.98	12.69	1.77	72.48	86.90
